# The Impact of Integration of Rapid Syphilis Testing during Routine Antenatal Services in Rural Kenya

**DOI:** 10.1155/2013/674584

**Published:** 2013-11-05

**Authors:** Eleanor Fleming, Jared Oremo, Katherine O'Connor, Aloyce Odhiambo, Tun Ye, Simon Oswago, Clement Zeh, Robert Quick, Mary L. Kamb

**Affiliations:** ^1^Centers for Disease Control and Prevention, National Center for HIV/AIDS, Viral Hepatitis, STD, and TB Prevention, Office of the Director, Office of Health Equity, 1600 Clifton Road Mailstop E07, Atlanta, GA 30333, USA; ^2^Division of Applied Sciences, Epidemic Intelligence Service, Centers for Disease Control and Prevention, Scientific Education and Professional Development Program Office, Atlanta, GA 30333, USA; ^3^Safe Water and AIDS Project, Kisumu 40100, Kenya; ^4^Division of Foodborne, Waterborne and Environmental Diseases, Centers for Disease Control and Prevention, National Center for Emerging and Zoonotic Infectious Diseases, Atlanta, GA 30333, USA; ^5^Centers for Disease Control and Prevention, Center for Global Health, Office of the Director, Atlanta, GA 30333, USA; ^6^Kenya Medical Research Institute (KEMRI), Kisian 40100, Kenya

## Abstract

We evaluated the integration of rapid syphilis tests (RSTs) and penicillin treatment kits into routine antenatal clinic (ANC) services in two rural districts in Nyanza Province, Kenya. In February 2011, nurses from 25 clinics were trained in using RSTs and documenting test results and treatment. During March 2011–February 2012, free RSTs and treatment kits were provided to clinics for use during ANC visits. We analyzed ANC registry data from eight clinics during the 12-month periods before and during RST program implementation and compared syphilis testing, diagnosis, and treatment during the two periods. Syphilis testing at first ANC visit increased from 18% (279 of 1,586 attendees) before the intervention to 70% (1,123 of 1,614 attendees) during the intervention (*P* < 0.001); 35 women (3%) tested positive during the intervention period compared with 1 (<1%) before (*P* < 0.001). Syphilis treatment was not recorded according to training recommendations; seven clinics identified 28 RST-positive women and recorded 34 treatment kits as used. Individual-level data from three high-volume clinics supported that the intervention did not negatively affect HIV test uptake. Integrating RSTs into rural ANC services increased syphilis testing and detection. Record keeping on treatment of syphilis in RST-positive women remains challenging.

## 1. Introduction

The World Health Organization (WHO) estimated that globally in 2008 1.4 million women had syphilis in pregnancy, causing approximately 520,000 adverse pregnancy outcomes including 305,000 perinatal deaths [1]. Untreated maternal syphilis currently equals or exceeds HIV, neonatal tetanus, or malaria as a cause of perinatal mortality [2]. Caused by the bacterium *Treponema pallidum*, syphilis is efficiently transmitted *in utero* to the fetus [3, 4]. When left untreated, adverse pregnancy outcomes can occur in up to 80% of affected pregnancies [4], with the most severe outcomes occurring in women with early (i.e., primary, secondary, or early latent) infections [3]. A recent meta-analysis adjusting for other causes of mortality estimated that, among asymptomatic women, untreated maternal syphilis caused stillbirth in 21% and neonatal death in 9% of affected pregnancies and led to low birth weight in 6% and congenital infection in 15% of surviving infants [5]. Infants born with syphilis may suffer disfigurement, delayed development, or other complications [3]. Early detection and adequate treatment (i.e., long-acting intramuscular penicillin) prevent poor outcomes due to mother-to-child transmission (MTCT) of syphilis [3, 4].

Nyanza Province, located in western Kenya, has the second highest neonatal (39 per 1000 live births) and fifth highest perinatal (37 per 1000 live births and stillbirths) mortality rates among Kenya's eight administrative provinces [6]. In comparison, Western European nations have neonatal and perinatal mortality rates of less than 5 per 1000 live births and 10 per 1000 live births plus stillbirths, respectively [7]. Nyanza also has the nation's highest prevalence of HIV (14%) and syphilis (2.3%) among reproductive-aged women [8].

Low maternal educational attainment, limited health care access, and poverty may adversely influence maternal and infant health outcomes. In 2008-2009, only 17% of women in Nyanza Province had completed primary school [6], 48% of pregnant women received antenatal care (ANC) at low-level facilities with minimal services, and 35% delivered their infants with a skilled birth attendant [6]. About 63% of the population lives on the equivalent of less than $1 a day. Although some ANC services, such as HIV testing, are provided without cost, women must pay for most basic services and supplies such as syphilis testing, antibiotics, and exam gloves.

Antenatal syphilis testing and treatment are highly cost effective [9] and are recommended by WHO in the basic ANC package [10]. Until recently, diagnosis in asymptomatic populations (the case for most pregnant women infected with syphilis) has depended on serologic tests requiring basic laboratory capacity that is unavailable at most low-level facilities. Newer, inexpensive, and rapid syphilis tests (RSTs) have been developed that use finger-prick whole blood samples and can be done at low-level facilities, with results and treatment provided at the clinic visit. In this evaluation, we sought to identify the feasibility and challenges of integrating RSTs into ANC services in low-level health facilities in a remote, rural setting and to assess the impact of RSTs on diagnosis and treatment of syphilis in pregnant women. Because introduction of new services can affect existing services, we also sought to ascertain the impact of the introduction of RSTs on uptake of rapid HIV testing.

## 2. Methods

### 2.1. Implementation of Antenatal Program

In 2011–2013, a Kenyan nongovernmental organization called the Safe Water and AIDS Project (SWAP), the Kenya Medical Research Institute (KEMRI), the Kenyan Ministry of Public Health and Sanitation (MOH), and the US Centers for Disease Control and Prevention (CDC) conducted an evaluation of a pilot RST program that was incorporated into a project that integrated nurse training; health facility improvement; and safe water, hygiene, and nutritional interventions into antenatal care in low-level facilities in two rural districts (Mbita and Suba) in Nyanza Province, Kenya. This evaluation was a component of a larger project integrating improved ANC services and safe water practices in low-level health facilities in Mbita and Suba Districts [11]. The large project involved an intervention with three components: (1) special training of nurses on emergency obstetrical care, neonatal resuscitation, safe water and hygiene practices, and improved ANC services including congenital syphilis prevention; (2) diagnosis and treatment supplies for clinics, including hand-washing and drinking water stations, infant resuscitation equipment, RST kits, and penicillin and injection equipment for women with reactive syphilis tests; and (3) public health commodities (safe water storage containers, water treatment products, soap, protein-fortified flour at ANC visits, and clean delivery kits with exam gloves, sterile razor blades, and swaddling cloths) provided to pregnant women as incentives to attend ANC. This intervention was available at no cost to the estimated 8000 pregnant women receiving ANC services during the intervention period (from March 2011 to February 2012). 

### 2.2. Study Design

For this evaluation assessing the feasibility and challenges of RSTs in low-level facilities, we used several methodologies: (1) a before-and-after assessment without concurrent controls using summary statistics of syphilis testing uptake and treatment among women receiving ANC services in eight facilities; (2) an assessment of diagnosis and treatment commodity stock outs (i.e., occasions when supplies of RSTs and penicillin in the clinics had been consumed and not yet resupplied) using stock books from the eight facilities; (3) an assessment of syphilis and HIV testing uptake using individual, patient-level data from ANC registries at three high-volume facilities; and (4) an evaluation of intervention challenges from in-depth interviews of nurses and patients.

### 2.3. Study Setting

We worked with all 25 rural health facilities to test the impact of various incentives on the use of antenatal and delivery services, including syphilis testing. Because of time and resource constraints, we limited the RST evaluation to all eight clinics selected using the following criteria: (1) public (MOH) clinics; (2) feasible to reach within one day; and (3) maintained antenatal registries from 2010 to 2012. Hereafter, these clinics are referred to as “priority clinics.” They included three dispensaries, four health centres, and a subdistrict hospital (Table 1). Dispensaries are the lowest-level facilities in the health care system, providing simple outpatient and preventive services with basic supplies and a nurse. Managed by a clinical officer and staffed by two or more nurses and a pharmacist, health centres can provide simple ambulatory and preventive services (e.g., vaccines). The subdistrict hospital is similar to health centres but has a laboratory for simple tests (e.g., blood slides for malaria parasites and urine dip tests).

### 2.4. Implementation of RSTs

In February 2011, faculty from Emory University, CDC, and SWAP provided a two-day, hands-on training for nurses and midwives that included modules on the public health importance, diagnosis, and treatment of congenital syphilis. Nurses were instructed on integrating RSTs with existing rapid HIV testing, counseling women about syphilis results, referring partners for treatment, and recording results of testing and treatment into ANC registries. Nurses began implementing enhanced services in March 2011.

### 2.5. Data Collection

For the before-and-after assessment comparing syphilis testing uptake, seropositivity, and treatment rates, we abstracted data from the records of all ANC attendees in the ANC registries at the eight facilities during the preintervention period (March 2010–February 2011) and during the intervention period (March 2011–February 2012). To further estimate syphilis treatment adherence, we reviewed ANC registry and stock book data to determine the ratio of penicillin vials used overall to the number of women whose syphilis tests were seroreactive. To assess the impact of syphilis testing on HIV testing rates, we used individual-level program data on syphilis and HIV testing from 330 consecutive women attending ANC at three “high-volume” clinics (i.e., served more than 400 women per year) during a time without shortages (“stock-outs”) of HIV or syphilis test kits. We collected individual data on gestational age, syphilis testing, HIV testing, and test results and determined syphilis and HIV test uptake by month before and during the intervention. We believed these data were useful because they allowed a purer assessment of test uptake in a time without test-kit stock-outs, and the individual-level data allowed exclusion of women with previous positive HIV status (i.e., ineligible for another HIV test). Finally, to assess the challenges of introducing RSTs, we conducted a series of qualitative, semistructured interviews with a convenience sample of nurses and mothers from the priority clinics. Interview guides were written in English and translated into the local language (Dholuo) by two SWAP enumerators who conducted and recorded the interviews.

### 2.6. Data Analysis

Quantitative data were entered into Microsoft Excel 6 databases and analyzed with SAS 9.3 (Cary, N.C.) and OpenEpi Version 2.3.1 (Atlanta, GA) to calculate the proportion of women tested for syphilis and HIV at the first ANC visit and compare the proportion testing positive in the 12-month intervals before and during the intervention. The ratio of penicillin vials used to total positive syphilis tests was also calculated. Qualitative data were analyzed using QSR International NVivo 8.0 (Melbourne, Australia). In an iterative process, we read and reread the interview texts for emerging themes and attached codes to the text representing them until no new themes emerged [12].

### 2.7. Ethical Review

The KEMRI Ethical Review Committee approved the study protocol (protocol 1898). The CDC Institutional Review Board approved the reliance on a non-CDC IRB in accordance with 45 CFR 46.114 (protocol 5996). Written informed consent was obtained from all participants, and personal identifiers were irretrievably removed from databases at the end of the study. 

## 3. Results

Of 1614 ANC attendees recorded in the ANC registries in the eight clinics during the one-year intervention period, 1123 (70%) were tested for syphilis compared with 279 (18%) of 1586 ANC attendees recorded in ANC registries during the prior year (*P* < 0.001) (Table 1). The increase in syphilis testing was statistically significant for each facility. Notably, at the three dispensaries, 79% of attendees were tested during the intervention period compared with none during the previous year. During the intervention period, 35 women (3%) tested positive for syphilis compared with one (<1%) during the previous year. No syphilis treatment data were collected in the ANC registries.

A total of 34 penicillin treatment kits were used at seven health facilities where 28 ANC syphilis tests were positive for a ratio of 1.2 : 1, suggesting that women testing positive received at least one penicillin injection (stock book (i.e., treatment) data in one facility were incomplete and thus were excluded). During the intervention period, most clinics had sufficient syphilis testing and treatment commodities. However, we noted that one dispensary temporarily lacked saline to dissolve penicillin and referred two patients to other facilities for treatment. Another facility experienced stock outs for one-two days during three separate months. During the 12-months prior to the intervention, syphilis test kits and treatment were unavailable at most low-level facilities and inconsistently available in the district hospitals. In contrast, HIV test kits were more consistently available at ANC facilities than syphilis tests during the 12 months prior to the intervention, although stock-outs in HIV test kits occurred for short periods during the intervention.

ANC registry data indicated that 1292 (72%) of 1614 ANC attendees had HIV testing during the intervention period compared to 1386 (87%) of 1586 attendees during the prior year (*P* < 0.001) (Table 1); HIV testing frequency during the intervention period was significantly lower at two of three dispensaries, two of four health centres, and the subdistrict hospital. The summary ANC registry data did not indicate results of previous HIV testing (previously positive women would not have been offered another HIV test).

**Figure 1:**
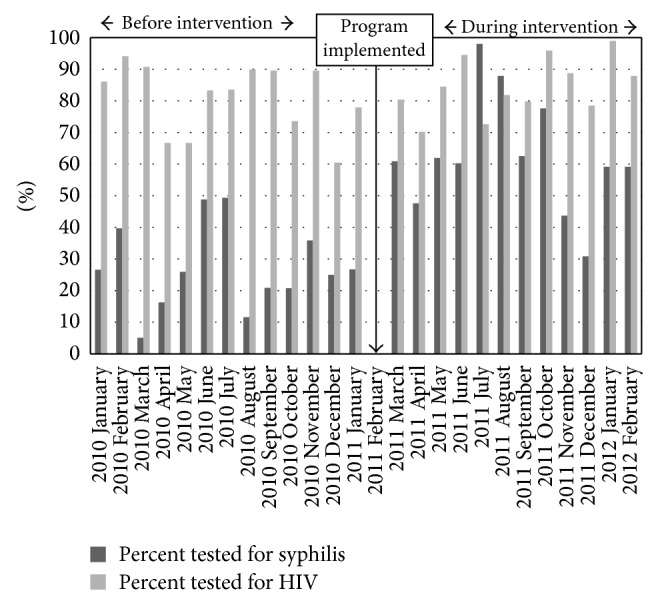
Percentage of pregnant women receiving syphilis and HIV tests at first ANC visit in three high-volume, rural health facilities Facilities evaluated included two health centres (facilities 4 and 7) and one subdistrict hospital (facility 8). 12 months before and 12 months during implementation of a rapid syphilis testing intervention.

Of 330 women attending ANC in three high-volume clinics during the intervention (combined data from two health centres and one subdistrict hospital), more women were tested for HIV (87%) than syphilis (76%); 67% received both tests and 4% received neither (Table 2). In the 12 months before intervention, HIV test uptake (60–94% per month, median 82%) exceeded syphilis test uptake (5–49% per month, median 26%). During the intervention, HIV uptake was similar (70–95% per month, median 82%), and syphilis uptake increased greatly (44–98% per month, median 60%). HIV testing exceeded syphilis testing during all months of the intervention period except for July-August 2012 (Figure 1).

From qualitative interviews, 10 nurses worked an average of 13 years (range, 2 years to 27 years) in a variety of facilities and four years at the current facility (range, one month to 10 years). Although syphilis treatment practices varied among the nurses, none of the nurses mentioned that patients are declining treatment because of partner violence. About half of the nurses (6) had attended the training on syphilis testing and treatment. Although all knew that proper treatment included intramuscular penicillin injections, not all knew the correct number of injections. Most nurses reported that the overall program had been successful but identified three main challenges. First, partner treatment was unaffordable for many; nurses recommended providing free partner treatment. Second, nurses were concerned about sustaining the program of free testing and treatment when the study ended. Finally, some nurses did not receive the training because of staff shortages at their facilities and recommended making training available to all nurses. Interviews with 21 mothers revealed that they were not always fully informed about the tests and did not feel they could ask nurses for more information. Testing practices were inconsistent from one pregnancy to the next and some mothers had never been tested.

## 4. Discussion

Findings from this evaluation suggest that RSTs are feasible for use in ANC services at low-level, rural facilities, resulted in an increased diagnosis and treatment of syphilis in pregnant women, and did not impact rapid HIV testing uptake. The percentage of pregnant women tested for syphilis at their first ANC visit increased dramatically from <1% to 70%, resulting in the identification of 35 infections. Although documentation of treatment was poor, the number of syphilis treatment kits used in the clinics roughly corresponded to the number of cases identified, suggesting that women testing positive were usually treated, likely averting cases of congenital syphilis. Syphilis prevalence in this population (3.5%) exceeded the rate reported in national surveys (2.3%), a finding that is consistent with studies elsewhere reporting higher syphilis prevalence among rural than urban women [13]. Syphilis testing is frequently unavailable in remote settings where syphilis infections may go undetected and consequently untreated. 

Although ANC registry data suggested that most women testing positive for syphilis received at least a single dose of 2.4 million units long acting penicillin intramuscularly (IM) (sufficient to prevent MTCT of syphilis), data recording was inconsistent and therefore difficult to interpret. Additionally, although a single IM dose of penicillin can prevent congenital syphilis, Kenya's guidelines (and global guidelines) recommend three weekly doses of 2.4 million units (7.2 million units total) to treat an asymptomatic, seroreactive mother with unknown duration of infection. Thus our data suggested that the syphilis-positive women were undertreated. Prior to the intervention, high-level approvals were obtained to modify existing ANC registries to include syphilis treatment; and the nurses' training involved intensive instruction on recommended treatment, the need to use IM penicillin during pregnancy, and practice on modifying the registry and documenting syphilis treatment. The failure of this aspect of the interventions suggests that to improve collection of syphilis treatment data, an important indicator of program effectiveness, MOH officials will need to add a syphilis treatment field to existing ANC registries and assure that providers are trained in both adequate treatment of syphilis in pregnancy and in entering treatment data properly [4]. 

Our data suggested that women were willing to be tested for both syphilis and HIV. Although overall ANC data suggested that a smaller percentage of women were tested for HIV during the intervention period than before, a more in-depth analysis of three high-volume clinics indicated the reverse: that, following the introduction of RSTs, HIV testing did not change. The latter finding was consistent with other studies that found that the introduction of RSTs did not adversely affect antenatal HIV test uptake [14]. The discrepant findings in this study could be explained by a lack of data on previous HIV testing in ANC registries. Women who previously tested positive for HIV would not be retested at later pregnancies, and including them in the denominators would (incorrectly) suggest a decrease in HIV test uptake. Furthermore, in November 2011 one of the tests used in Kenya's HIV testing algorithm was removed from stocks due to inadequate performance in some lots [15, 16], leading to short-term but widespread shortages in HIV tests. This may explain the documentation of HIV test shortages in some clinics during the intervention period. Some ANC attendees in early 2012 may have been offered HIV testing at later ANC visits or not at all.

The qualitative data suggested three problems in service delivery that are modifiable. First, the nurses did not adequately communicate the importance of syphilis testing and treatment to ANC attendees, and women did not feel comfortable asking nurses for information. This problem may have been exacerbated by the frequent transfer of nurses and other providers who may have taken their knowledge of performing RSTs, maintaining RST stock, and recording results with them without transferring knowledge to new nurses. Addressing these training problems, as well as possibly including community outreach regarding congenital syphilis prevention, could help improve program operation. Second, treatment of male partners in this program was unsuccessful. Their lack of participation in ANC and inability to pay for treatment is a lost opportunity that also places treated women at risk of reinfection. The ANC setting has been found to be an appropriate place to counsel and test male partners in other programs (e.g., HIV) [17, 18], and treatment of partners for syphilis would be a relatively simple and inexpensive addition, possibly serving as an incentive for men to accompany their partners to ANC. Finally, some nurses were concerned about the sustainability of the program when outside funding to purchase RSTs is no longer available. Health ministries will need to consider means of covering basic ANC services in order to achieve global maternal and infant health goals.

Our evaluation had several limitations. First, because the facilities we evaluated were in only two districts of Nyanza Province they may not have been representative of rural facilities elsewhere. Second, most antenatal registries were incomplete, limiting our interpretation of the findings, particularly of treatment of women with positive RSTs. Despite this limitation, the very large increment in the percentage of women receiving RSTs left little doubt about the program's impact on syphilis detection. Third, HIV test uptake in the 8 clinics was likely underestimated because antenatal registries did not allow identification of previously positive women. Additionally, the removal of one of the HIV tests in Kenya's HIV testing algorithm during the intervention may have led to lower HIV testing rates in some clinics that were unrelated to introduction of RSTs (a problem inherent to before-and-after studies). Finally, the perspectives from the key informants may not have represented the broader population of nurses and mothers. However, we believe the sample was of sufficient size to capture a comprehensive range of perspectives.

In conclusion, our findings suggest that integrating RST into routine ANC services in low-level facilities in remote, rural settings where many women in sub-Saharan Africa receive MCH services was feasible, led to large increases in syphilis testing uptake, and uncovered higher syphilis prevalence than had been previously reported. These data are important for health ministries seeking practical and affordable methods for providing syphilis testing, a recommended component of basic ANC services. The data also add support for projected benefits from modeling studies indicating that early syphilis detection and treatment in antenatal women are a cost-saving intervention in settings with moderate-to-high syphilis prevalence, even when laboratory capacity is limited [9]. Introducing RSTs also did not appear to adversely impact HIV testing in the ANC setting [14]. This study documented that reporting treatment data was not optimal but could be addressed by improving ANC registries and training providers. Additional training for nurses could help ensure that patients are fully counseled on the tests that are offered to them and on the meaning of the results. Nurses could also receive additional training on partner notification to ensure that partners of patients diagnosed and treated are also tested and treated. Community education programs would be another strategy to promote syphilis testing to mothers and their partners. Taken together, these strategies could provide a meaningful contribution to global efforts to reduce MTCT of syphilis.

## Figures and Tables

**Table 1:** Antenatal syphilis and HIV testing coverage and seropositivity rates at eight priority clinics, 12 months before and 12 months during implementation of a rapid syphilis testing intervention, Suba and Mbita Districts, Kenya, March 2010–February 2012.

Clinic type	Before intervention					During intervention						
	# at 1st ANC	# (%) Tested for syphilis	# (%) Tested for positive syphilis	# (%) Tested for HIV	# (%) Tested positive for HIV	# at 1st ANC	# (%) Tested for syphilis	# (%) Tested for positive syphilis	# (%) Tested for HIV	# (%) Tested positive for HIV	Change in syphilis testing (*P* value)	Change in HIV testing (*P* value)
Dispensary												
Facility 1	34	0	0	33 (97)	15 (46)	70	66 (94)	7 (11)	58 (83)∗	24 (41)	<0.001	0.04
Facility 2	162	0	0	153 (94)	28 (18)	171	132 (77)	6 (5)∗∗	162 (95)	22 (14)	<0.001	0.45
Facility 3	157	0	0	151 (96)	22 (15)	122	90 (74)	2 (2)	92 (75)∗	9 (7)	<0.001	<0.001
Subtotal	**353**	**0**	**0**	**337 (95)**	**65 (19)**	**363**	**288 (79)**	**15 (5)**	**312 (86)**	**55 (31)**	**<0.001**	**<0.001**

Health center												
Facility 4	351	104 (30)	0	307 (88)	40 (13)	345	161 (47)	7 (4)	300 (87)	34 (11)	<0.001	0.84
Facility 5	114	3 (7)	0	97 (85)	11 (11)	144	115 (80)	3 (3)	126 (88)	10 (8)	<0.001	0.57
Facility 6	178	1 (1)	0	165 (93)	26 (16)	216	166 (77)	6 (4)	174 (81)∗	12 (7)	<0.001	<0.001
Facility 7	297	98 (33)	1 (1)	239 (81)	35 (15)	255	206 (81)	3 (2)	159 (62)∗	38 (13)	<0.001	<0.001
Subtotal	**940**	**206 (22)**	**1 (<1)**	**808 (86)**	**112 (14)**	**960**	**648 (68)**	**19 (3)**	**759 (79)**	**94 (12)**	**<0.001**	**<0.001**

Subdistrict hospital												
Facility 8	293	73 (25)	0	241 (82)	34 (14)	291	187 (64)	1 (1)	221 (76)∗	23 (10)	<0.001	0.03

Total	1586	279 (18)	1 (<1)	1386 (87)	211 (25)	1614	1123 (70)	35 (3)	1292 (72)	172 (15)	<0.001	<0.001

^1^Dispensaries: lowest level of health system, typically have one nurse and provide simple ambulatory and preventive services.

^2^Health centres: lower-level facilities that are larger than dispensaries, under a clinical officer and with basic health staff (e.g., two or more nurses, a pharmacist), and provide ambulatory and preventive health services (e.g., vaccines).

^3^Subdistrict hospital: similar to health centre but has basic laboratory services (although not RPR testing).

∗Facilities reporting HIV test stock outs from February 29–April 2012.

∗∗Facility reporting syphilis test kit stock outs for the following dates: June 7–9, 2011, September 7, 2011, and October 11-12, 2011, due to problems with distribution.

**Table 2:** Percentage of pregnant women tested for HIV, syphilis, or both, by clinic and gestational age, among consecutive women attending first antenatal care visit in three high-volume clinics, Suba and Mbita Districts, Kenya, from March 2011 to February 2012.

	HIV test only	Syphilis test only	Both tests	Neither test	Any HIV test	Any syphilis test
	*N* (%)	*N* (%)	*N* (%)	*N* (%)	*N* (%)	*N* (%)
Women at 1st ANC visit (*N* = 330)	66 (20.0)	29 (8.8)	222 (67.3)	13 (3.9)	288 (87.3)	251 (76.1)
Clinic						
Facility 4 (*N* = 100)	20 (20.0)	3 (3.0)	77 (77.0)	0	97 (97.0)	80 (80.0)
Facility 7 (*N* = 120)	6 (5.0)	10 (8.3)	100 (83.3)	4 (3.3)	106 (88.3)	110 (91.7%)
Facility 8 (*N* = 110)	40 (36.4)	16 (14.6)	45 (40.9)	9 (8.2)	85 (77.3)	61 (55.5)
Gestational age at 1st ANC∗						
≤12 weeks (*N* = 16)	4 (25.0)	3 (18.8)	9 (56.3)	0	13 (81.3)	12 (75.0)
13–24 weeks (*N* = 130)	21 (16.2)	9 (6.9)	95 (73.1)	5 (3.9)	116 (89.2)	104 (80.0)
weeks (*N* = 163)	37 (22.7)	15 (9.2)	108 (66.3)	3 (1.8%)	145 (89.0)	123 (75.5)

^*^21 records had missing information.
